# Analysis of cytokines in the aqueous humor during intravitreal Ranibizumab treatment of diabetic macular edema

**DOI:** 10.1038/s41598-021-03433-2

**Published:** 2021-12-14

**Authors:** Luiz Guilherme Azevedo de Freitas, David Leonardo Cruvinel Isaac, Murilo Batista Abud, Alexandre Dantas Soares Quintas Segundo, Mariana Larissa Alvino Barros, Gabriela Caroline Monteiro de Albuquerque, Bruna Dantas Aires Guimarães, Clarice Neuenschwander Lins de Morais, Marcos Pereira de Ávila

**Affiliations:** 1 Physician, Retina and Vitreous Department at the Ophthalmology Reference Center, Hospital das Clínicas, Federal University of Goiás—UFG, Goiânia, GO Brazil; 2 Physician, Santa Luzia Eye Hospital/Santa Luzia Foundation, Estrada Do Encanamento, 909, Recife, PE 52060-000 Brazil; 3 Virology and Experimental Therapy Laboratory, Aggeu Magalhães Research Center, Oswaldo Cruz Foundation, Recife, PE Brazil; 4 Professor and Head of the Ophthalmology Service of the Federal University of Goiás, Goiânia, GO Brazil; 5 Head of the Retina and Vitreous Department of the UFG, Goiânia, GO Brazil

**Keywords:** Biochemistry, Biological techniques, Anthropology, Eye manifestations

## Abstract

This study aimed to analyze the concentrations of VEGF, b-FGF, TNF, interleukin (IL)-1, IL-6, IL-8, IL-10, and IL-12 in the aqueous humor of patients with diabetic macular edema with and without peripheral retinal ischemia and to ascertain the changes in the levels of these molecules during treatment with ranibizumab. A therapeutic, prospective, randomized interventional study was carried out. Twenty-four eyes from 24 patients were studied and divided into 3 groups. Group 1 (9 eyes) included patients with diabetic macular edema without peripheral ischemia. Group 2 (10 eyes) included patients with diabetic macular edema with peripheral ischemia. Group 3 (5 eyes), the control group, included patients without systemic and/or eye diseases. Patients in Groups 1 and 2 received 3 intravitreal injections of 2 mg/0.05 ml ranibizumab at an interval of approximately 30 days. Before administering the injections, the aqueous humor was collected. In the control group, aqueous humor was collected before facetectomy. During treatment, the median IL-6 concentration significantly increased in Group 1 but showed a slight but not significant decrease in Group 2. Interleukin 8 levels were significantly different at the end of treatment compared to the beginning in Groups 1 and 2. TNF, IL-1, IL-10, and IL-12 levels were practically unchanged in both groups. VEGF was significantly reduced at the end of the study in Groups 1 and 2. B-FGF was not detected in most of the studied patients, and in those with detectable levels, there was no significant variation. There was a significant increase in the median level of interleukin 6 in the group without ischemia and a significant decrease in VEGF in both groups. The cytokines TNF, IL-1, IL-10, and IL-12 did not show significant variation.

## Introduction

Diabetic retinopathy (DR) is one of the main causes of vision loss worldwide^1^.

Macular edema (ME) is considered the main cause of visual impairment in patients with diabetic retinopathy and can appear at all stages of the disease, affecting approximately 30% of diabetic patients who have lived with the disease for more than 20 years^2–5^.

Tests such as optical coherence tomography (OCT) and fluorescein angiography (FA) are performed for the diagnosis and monitoring of patients with DR^6^.

Fluorescein angiography is essential for acquiring information about the disease stage^7,8^. New devices called wide-angle fluorescein angiography can capture images with angles between 55° and 200°^9^. This new technology has aroused interest in analyzing the retinal periphery and correlating observed changes with the emergence and progression of diabetic retinopathy^9–12^.

We believe that regions of poor perfusion stimulate the production of vascular endothelial growth factor (VEGF) and that high VEGF levels in the vitreous induce ME^5,13–15^.

VEGF is increased in patients with diabetic retinopathy. Cytokines such as interleukins, TNF, and b-FGF are also altered in this disease^16–22^.

We performed an unprecedented study in which we recruited patients with diabetic macular edema with and without peripheral retinal ischemia for an analysis and comparison of the concentrations of the molecules IL-1, IL-6, IL-8, IL-10, IL-12, TNF, VFGF, and b-FGF during intravitreal treatment with ranibizumab.

## Methods

All methods were performed according to relevant guidelines.

The consent form was signed by all research participants or legal guardians.

The samples were collected between April 2018 and March 2019.

We performed optical coherence tomography with the Heidelberg Spectralis® HRA + OCT platform to diagnose edema and angiography with a noncontact lens using the Ultra-Widefield Spectralis® module at a 102° angle to detect the presence of peripheral retinal ischemia. Individuals who presented areas of ischemia with more or less than a 5-disc diameter area were included in the ischemia group.

The study was approved by the Ethics Committee of the Medical Research Ethics Council of Hospital Agamenon Magalhães, Recife, Pernambuco.

In the present study, patients were recruited from the Unified Health System. These patients usually do not have a real understanding of the disease or recognize that treatment must be continuous to be effective, even after receiving all our instructions.

These circumstances and other facts have a considerable negative impact on patient recruitment and adherence to the study protocol. Therefore, the resulting relatively small sample renders it unfeasible to conduct a multivariate analysis with robust results.

### Study population and design

This was a therapeutic, prospective, randomized interventional study. The sample consisted of 24 eyes of 24 patients divided into 3 groups: Group 1 (9 eyes), DME without peripheral retinal ischemia; Group 2 (10 eyes), DME with peripheral retinal ischemia; and Group 3 (5 eyes), control (no systemic or eye diseases).

**Figure 1:**
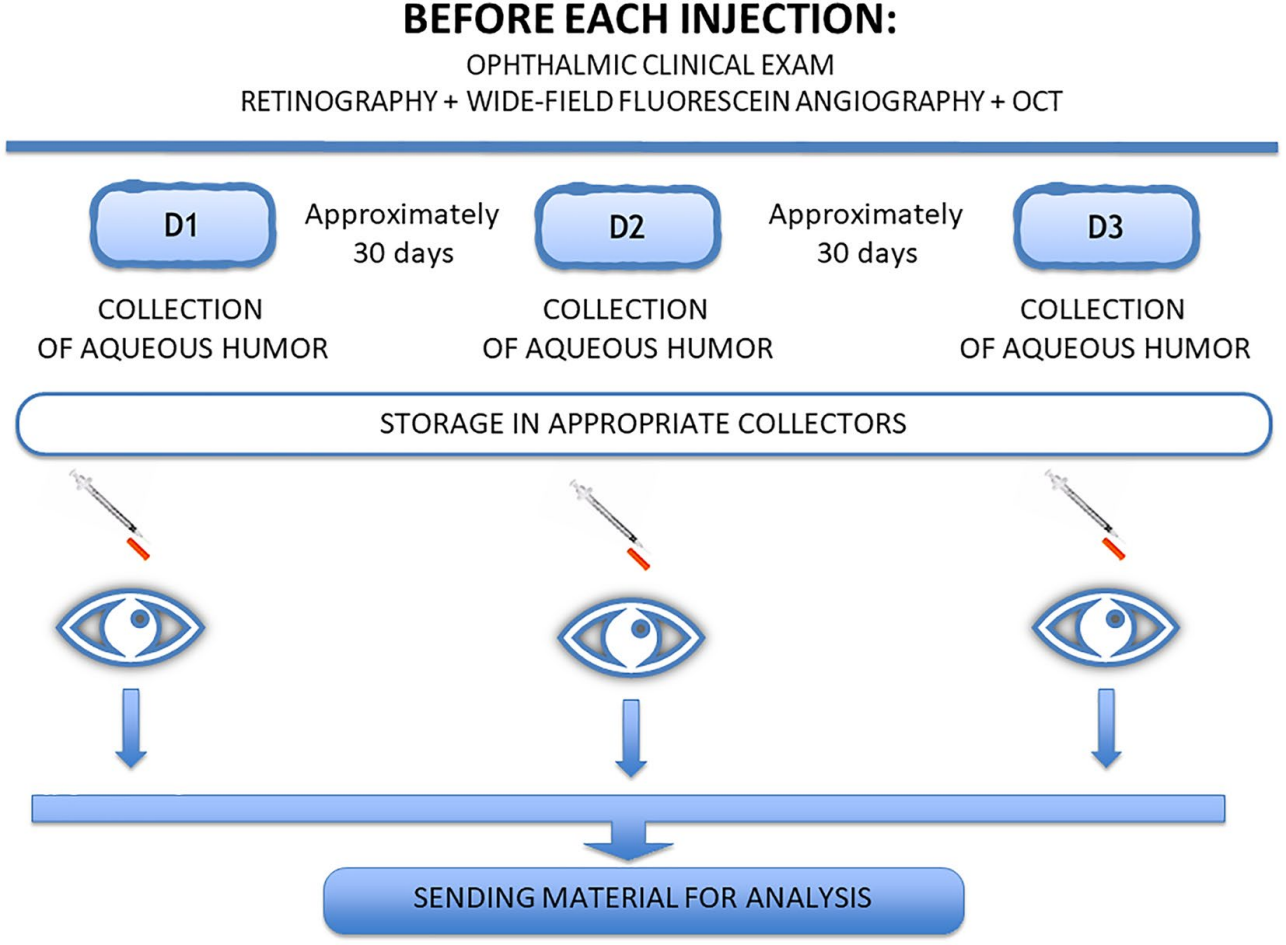
Study design.

Patients in Groups 1 and 2 received 3 intravitreal injections of ranibizumab at an interval of approximately 30 days. Before each treatment injection, we collected aqueous humor samples (Fig. 1). For Group 3, samples were collected on the day of cataract surgery.

### Analysis of cytokines

The Human Inflammatory Cytokine kit (BD) kit was used to analyze interleukins 1, 6, 8, 10, and 12 and TNF, and the CBA Flex Set (BD Medical) was used to evaluate human VEGF and bFGF.

### Evaluation of results and statistical analysis

In this study, data were previously evaluated to determine the need for parametric or nonparametric approaches. Thus, the variables included in the study were tested for distribution of normality and homogeneity using the Shapiro–Wilk and Bartlett tests, respectively.

A significance level of 5% was applied. We used R software (R DEVELOPMENT CORE TEAM, 2016) to evaluate the results of the study.

### Inclusion criteria

The inclusion criteria were as follows: diabetic macular edema diagnosed by wide-angle fluorescein angiography exams and optical coherence tomography; age at least 18 years; and no previous treatment with laser photocoagulation and no treatment with antiangiogenic drugs in the past 3 months.

### Exclusion criteria

The exclusion criteria were as follows: cataract patients with corneal or vitreous opacities that prevent adequate visualization of the retina; patients with macular edema not due to diabetic retinopathy; patients who underwent previous vitreoretinal surgery in the eye to be studied; and refusal to participate in the study.

### Clinical and complementary examinations

Patients underwent ophthalmic clinical evaluations. Visual acuity exams were performed with better correction by ETDRS, applanation tonometry, biomicroscopy, and indirect binocular ophthalmoscopy.

Optical coherence tomography and fluorescein angiography examinations were performed using the Heidelberg Spectralis® HRA + OCT platform. A noncontact lens was used in the Ultra-Widefield Spectralis® module to capture the 102° wide-field angiographic image.

### Collection and analysis of aqueous humor

Limbal paracentesis was performed with a 30-gauge needle attached to a 1-ml syringe. The collected sample volume ranged between 0.10 ml and 0.3 ml.

We quantified cytokine levels using the Cytometric Bead Array (CBA) system following the manufacturer's instructions (BD Biosciences, USA). The cytokines IL-1, IL-6, IL-8, IL-10, IL-12, and TNF were analyzed using the Human Inflammatory Cytokine kit (BD), and human VEGF and b-FGF were analyzed using the CBA Flex Set from BD.

### Ethical approval

This study was approved by the Ethics Committee of the Medical Research Ethics Council of Hospital Agamenon Magalhães, Recife, Pernambuco (Number: 2.540.686).

### Informed consent

Informed consent was obtained from all individual participants included in the study.

## Results

The number of participants in each group was small, which somewhat limited the results obtained.

Studies with a larger number of patients should be conducted to obtain better data with greater fidelity.

### Group 1

Group 1 included 9 patients (4 males and 5 females) aged 52–77 years (± 9.34) with diabetic macular edema without peripheral retinal ischemia; these patients were treated monthly with ranibizumab.

#### Visual acuity and macular thickness

**Figure 2:**
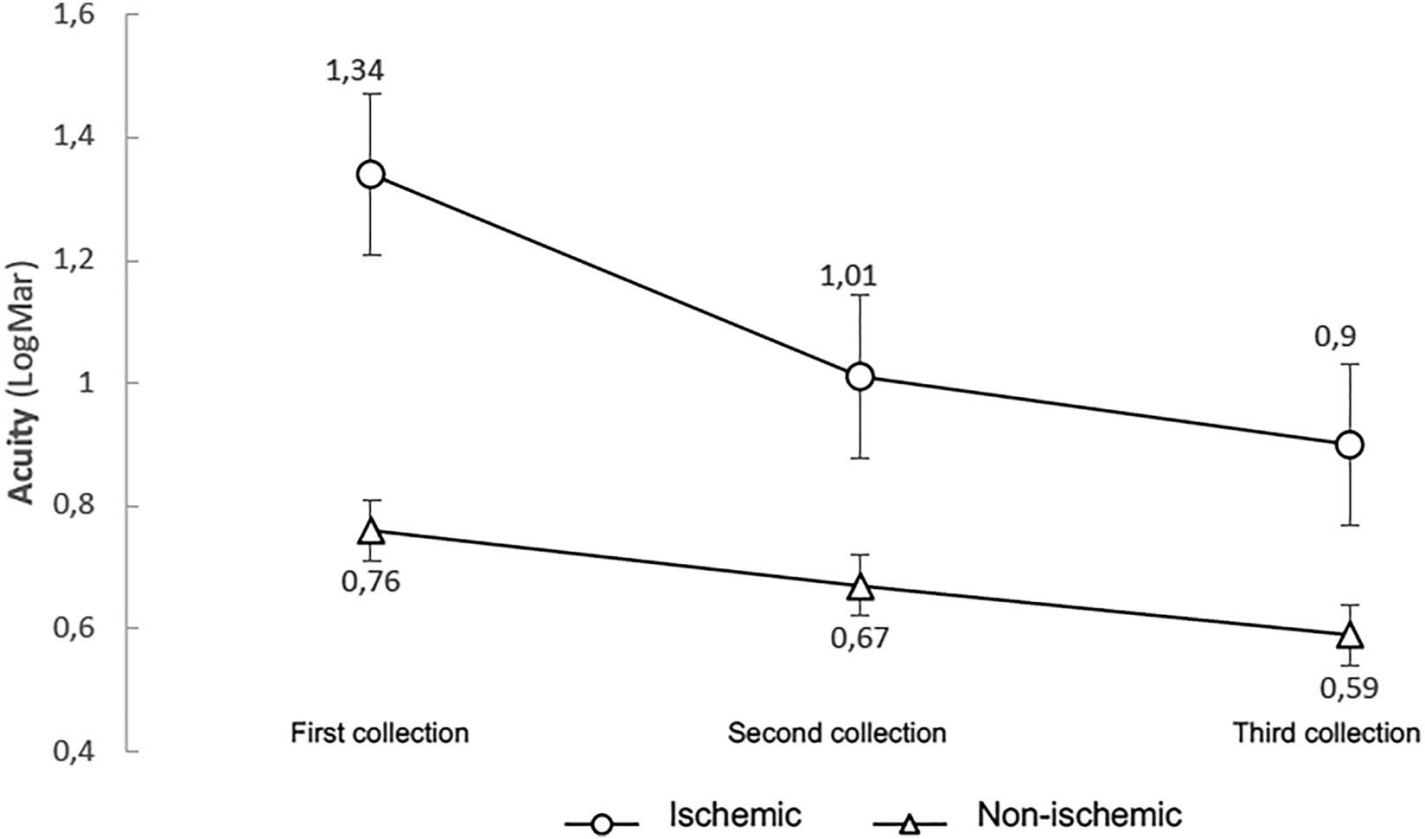
Comparison of the variation in visual acuity in the nonischemic and ischemic groups.

At the end of the study, 8 patients showed an improvement in visual acuity, and one patient showed no visual improvement but maintained his or her initial vision (Fig. 2).

**Figure 3:**
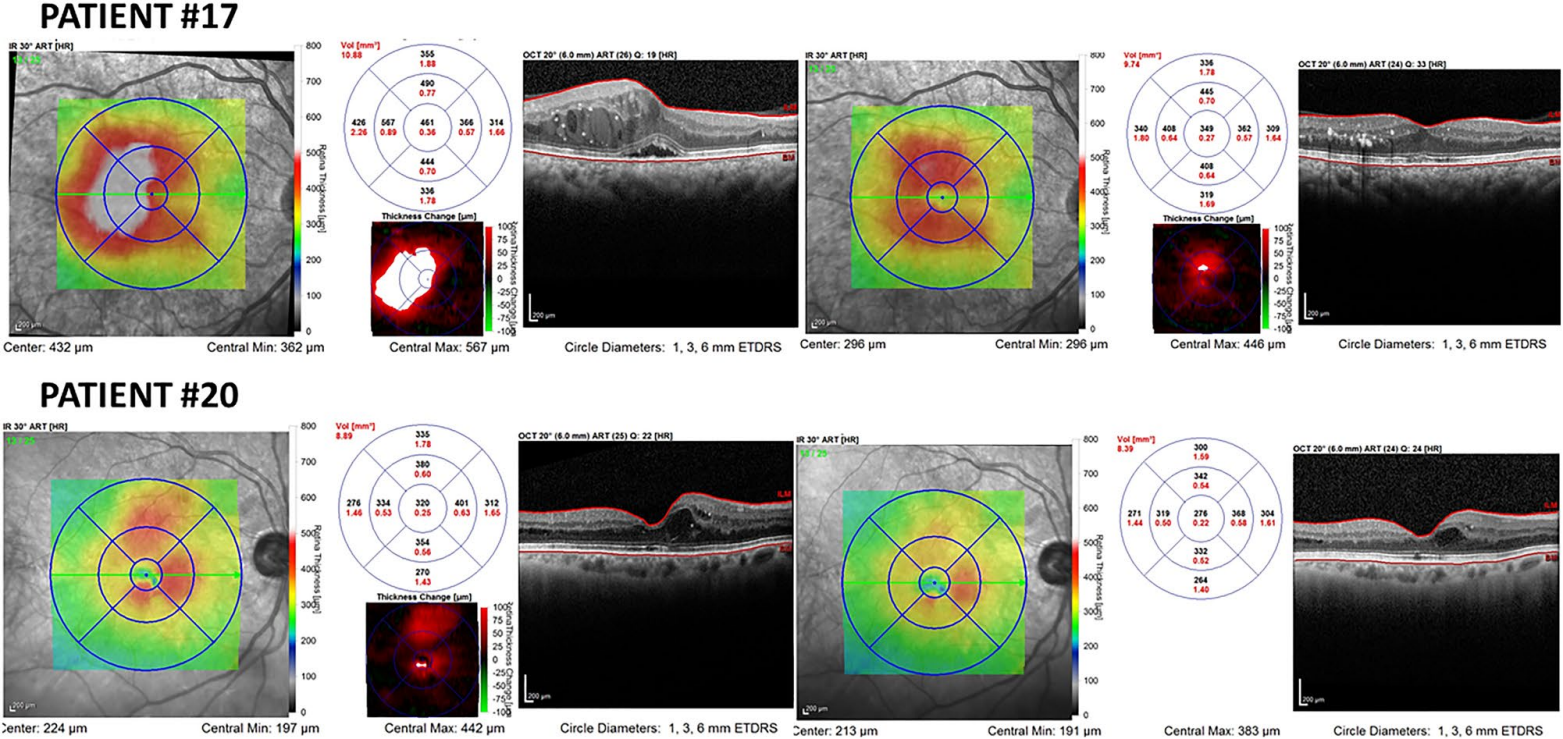
OCT showing a reduction in macular edema after treatment in Group 1.

Upon OCT examination, all patients presented a reduction in central macular thickness upon analysis of the macular thickness map, as illustrated in two patients (Fig. 3).

#### Clinical examinations and fluorescein angiography

Upon biomicroscopy examination, one patient was diagnosed with pseudophakia, and 8 were phakic. No cataracts formed on any lens during the study.

At gonioscopy, neovessels at the angle or iris were not observed before or at the end of the study.

Upon examination by angiography, no patient presented vascular changes in terms of staining type or retinal neovascularization during the treatment period.

### Group 2

Group 2 included 10 patients (7 males and 3 females) aged 47–68 years (± 7.06) with DME with peripheral retinal ischemia; these patients were treated monthly with ranibizumab.

#### Visual acuity and macular thickness

At the end of the study, 8 patients showed an improvement in visual acuity. Those who did not improve maintained their initial visual acuity (Fig. 2).

**Figure 4:**
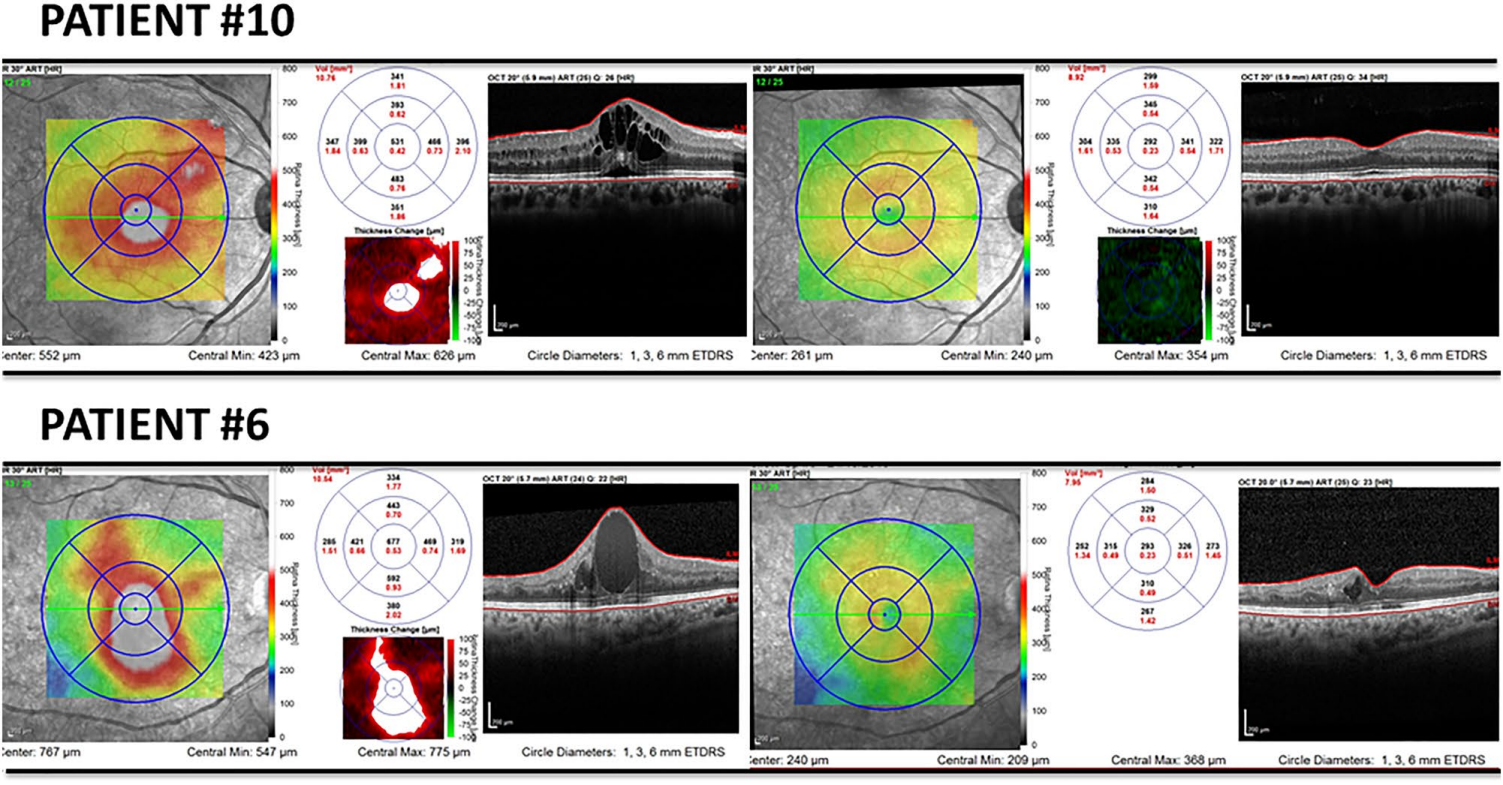
OCT showing reduction of macular edema after treatment in Group 2.

Upon OCT examination, all patients presented a reduction in central macular thickness upon analysis of the macular thickness map, as illustrated in two patients (Fig. 4).

#### Clinical examinations and fluorescein angiography

Upon biomicroscopy examination, 9 patients were phakic. No cataracts developed on any lens during the study. At gonioscopy, neovessels at the angle or iris were not observed before and at the end of the study.

**Figure 5:**
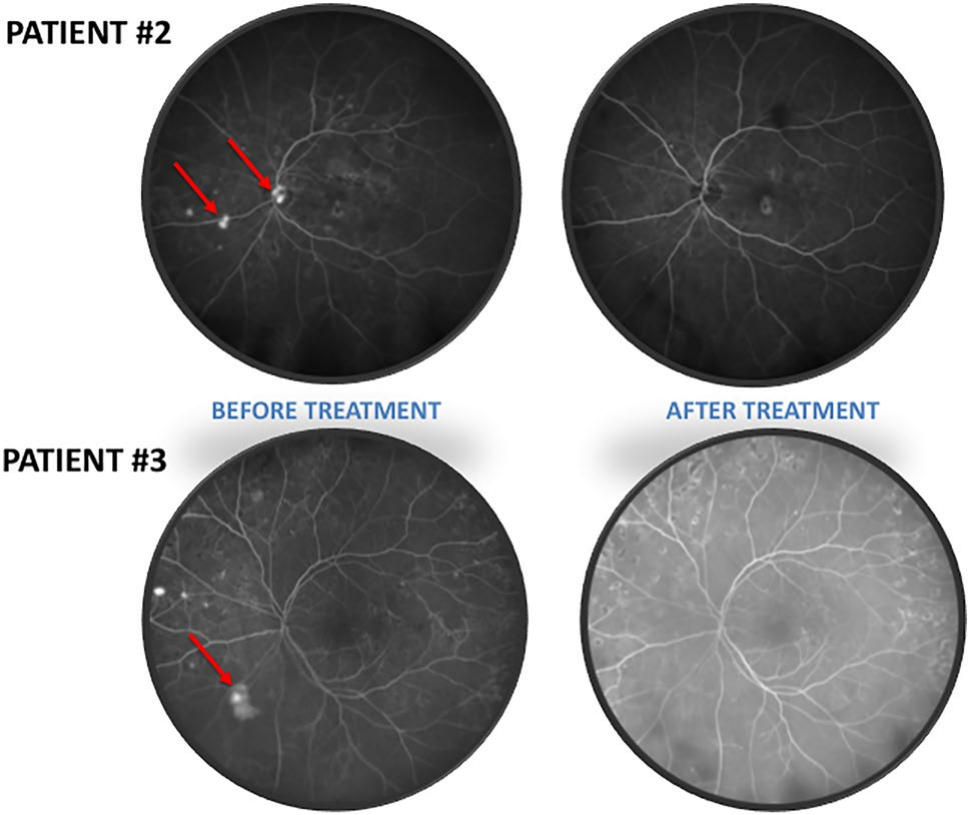
Angiography showing regression of the retinal neovessels (red arrow).

Angiography at the beginning of the study revealed retinal neovessels in two patients, and one of these patients also had disc neovessels at this timepoint. At the end of the study, both patients presented with regression of the new vessels (Fig. 5).

#### Analysis of cytokines in aqueous humor

The median values at the beginning and end of treatment were compared in Groups 1 and 2. For the control group, we used the values at the beginning of the treatment period (baseline levels) (Table 1).

**Table 1:** Values of medians and standard deviation of cytokines in aqueous humor in the initial time of treatment (D1) and after treatment (D3), for the groups: non-ischemic and ischemic and values of the medians of the control group at baseline treatment. D1: First collection; D3: Third Collection.

Cytokine	Groups	Median in D1	Standard deviation	Median in D3	Standard deviation
IL12	1—Control	3.4	0.18		
	2—Without ischemia	4.21	0.79	2.88	1.60
	3—Ischemia	3.6	0.44	3.67	0.46
IL06	1—Control	8.23	3.33		
	2—Without ischemia	10.78	4.09	26.35	19.56
	3—Ischemia	28.02	20.31	27.41	211.62
IL08	1—Control	4.66	3.21		
	2—Without ischemia	13.18	3.95	18.15	11.94
	3—Ischemia	15.43	10.65	20.80	19.73
IL10	1—Control	5.77	0.31		
	2—Without Ischemia	5.62	3.95	18.15	11.94
	3—Ischemia	5.71	10.65	20.80	19.73
IL1-1	1—Control	0	25.52		
	2—Without ischemia	1.61	1.55	0.00	2.53
	3—Ischemia	0.81	0.85	0.56	0.92
TNF	1—Control	3.8	0.21		
	2—Without Ischemia	4.54	1.09	3.16	1.98
	3—Ischemia	3.86	0.48	3.87	0.47
VEGF	1—Control	132.54	99.94		
	2—Without ischemia	170.04	120.54	0.00	57.23
	3—Ischemia	174.73	142.91	0.00	0.00
b.FGF	1—Control	0	0		
	2—Without ischemia	0	10.53	0.00	0.00
	3—Ischemia	0	0	0.00	0.00

The values in the control group were as follows: IL-1: 0 pg/ml, IL-6: 8.23 pg/ml, IL-8: 4.66 pg/ml, IL-10: 5.57 pg/ml, IL-12: 3.4 pg/ml, TNF: 3.8 pg/ml, VFGF: 132.54 pg/ml, and b-FGF: 0.00 pg/ml (Table 1).

Upon analysis of the variation in the median IL-6 level during treatment, we observed a significant increase in Group 1 at the end of the study (10.78–26.35 pg/ml, p = 0.0148).

**Figure 6:**
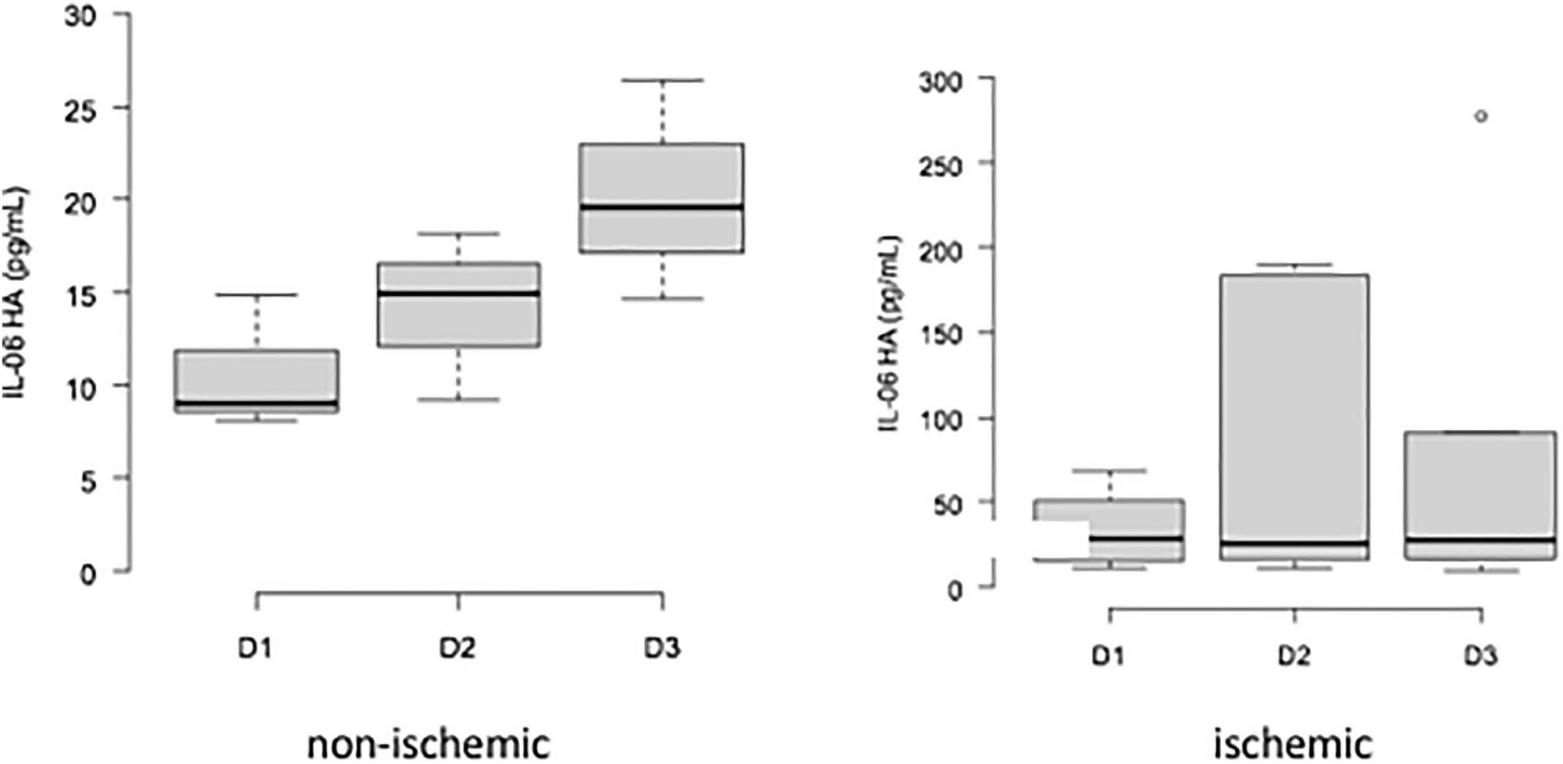
Variation in the median IL-6 level during treatment in Groups 1 and 2. D1: first collection; D2: second collection; D3: third collection.

In Group 2, there was a slight decrease in the median IL-6 concentration, but the difference was not statistically significant (28.02–27.41 pg/ml, p = 0.194) (Fig. 6).

**Figure 7:**
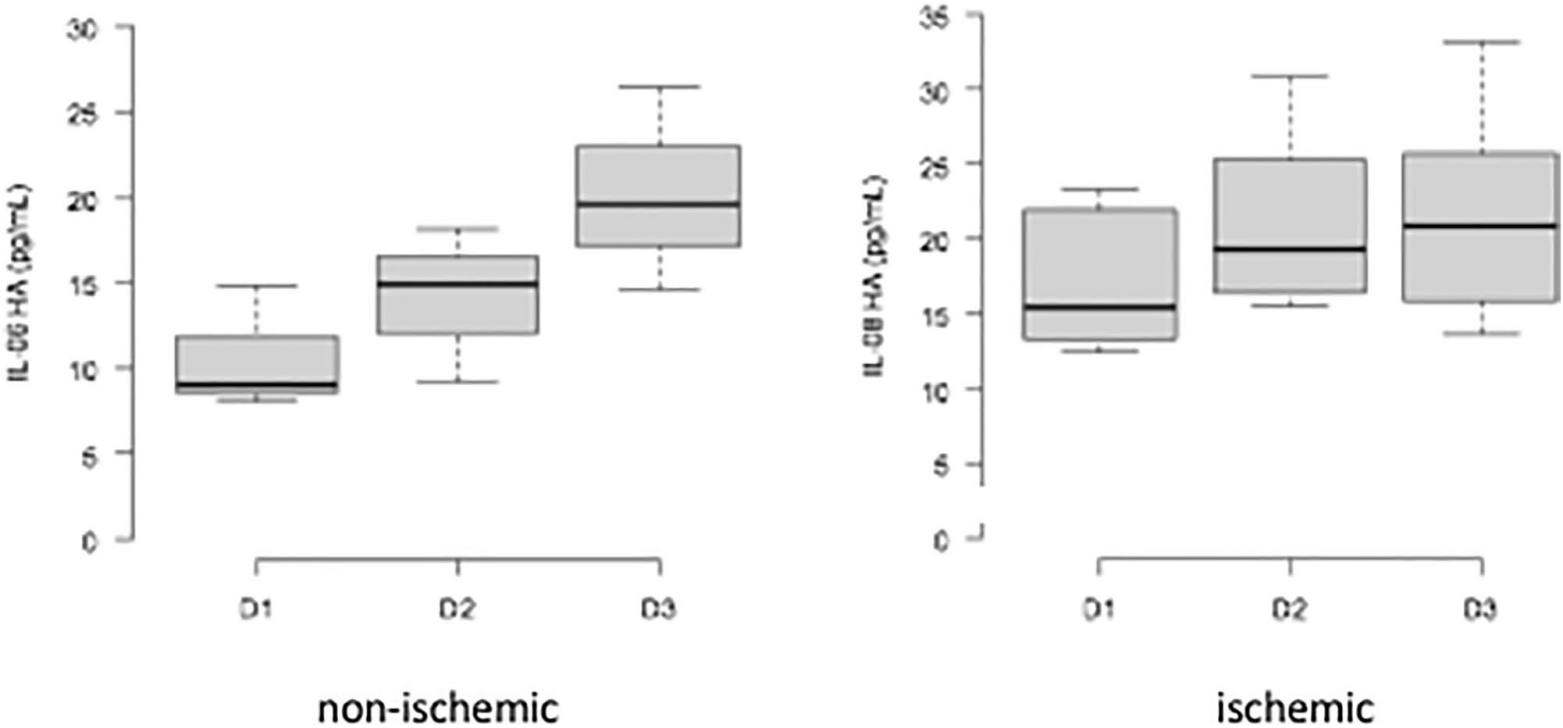
Variation in the median IL-8 level during treatment in Groups 1 and 2. D1: first collection; D2: second collection; D3: third collection.

There were significant variations in IL-8 in Groups 1 and 2. The median IL-8 level increased from 13.8 ± 3.95 pg/ml to 18.15 ± 12.65 pg/ml in Group 1 (p = 0.0234) and from 15.43 ± 10.65 pg/ml to 20.8 ± 19.73 pg/ml in Group 2 (p = 0.037) (Fig. 7).

Groups 1 and 2 showed a reduction in the median IL-1 level, but these decreases were not statistically significant: Group 1, 1.61 ± 1.66 pg/ml to 0.0 ± 2.53 pg/ml, p = 0.4185; and Group 2, 0.81 ± 0.85 pg/ml to 0.56 ± 0.92 pg/ml, p = 0.441 (Table 2).

**Table 2:** Variation of medians and standard deviation of cytokines in aqueous humor in the initial time of treatment (D1) and after treatment (D3) for groups 1 and 2.

	Group without ischemia			Group with ischemia		
	Median variation	Standard deviation	p	Median variation	Standard deviation	p
IL-1	1.61–0.00	± 1.66/2.53	0.4185	0.81–0.56	± 0.85/0.92	0.441
IL-6	10.78–26.35	± 4.09/19.56	**0.0148**	28.02–27.41	± 20.3/211.62	0.194
IL-8	13.8–18.15	± 3.95/12.65	**0.0234**	15.43–20.8	± 10.65/19.73	**0.037**
IL-10	5.62–5.29	± 0.7/1.17	1.000	5.71–5.76	± 0.40/0.29	0.626
IL-12	4.21–2.88	± 0.79/1.6	0.425	3.60–3.67	± 0.44/0.49	0.489
TNF	4.54–3.16	± 1.09/1.98	1.000	3.86–3.87	± 0.48/0.47	0.155
VEGF	170.04–0.00	± 120.54/57.23	**0.003**	174.73–0.0	± 0/0	**0.002**
b-FGF	0	0	0	0	0	0

The concentrations of two interleukins, IL-10 and IL-12, were practically unchanged in both groups. IL-10 levels varied between 5.62 ± 0.70 pg/ml and 5.29 ± 1.17 pg/ml in Group 1 (p = 1.00) and between 5.71 ± 0.40 pg/ml and 5.76 ± 0.297 pg/ml in Group 2 (p = 0.626).

Regarding interleukin 12, the values were 4.21 ± 0.79 pg/ml and 2.88 ± 1.60 pg/ml in Group 1 (p = 0.425) and 3.60 ± 0.44 pg/ml and 3.67 ± 0.49 pg/ml in Group 2 (p = 0.489) (Table 2).

We did observe some changes in the median TNF concentration, although the values remained similar throughout the treatment period: Group 1, 4.54 ± 1.099 pg/ml and 3.16 ± 1.98 pg/ml (p = 0.155); and Group 2, 3.86 ± 0.48 pg/ml and 3.87 ± 0.47 pg/ml (p = 0.155) (Table 2).

**Figure 8:**
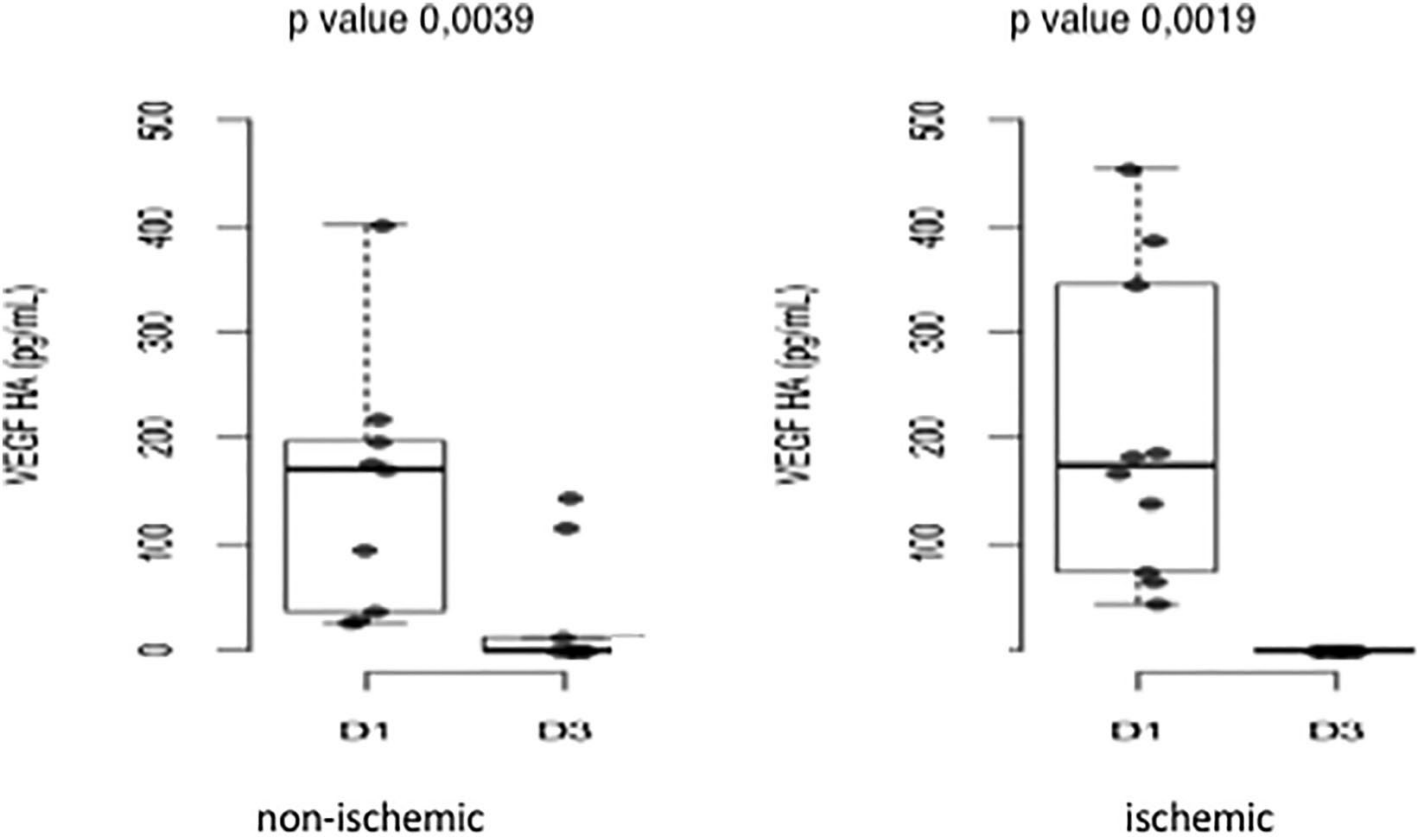
Variation in the median VEGF concentration before and after treatment in Groups 1 and 2. D1: first collection; D3: third collection.

The median VEGF concentration decreased significantly in both groups: Group 1, 170.04 ± 120.54 pg/ml and 0.0 ± 57.23 pg/ml (p = 0.0039); and Group 2, 174.73 ± 142.91 pg/ml and 0.00 ± 0.00 pg/ml (p = 0.0019) (Fig. 8).

b-FGF was detected in only 3 patients in Group 1 and none in Group 2. Thus, we could not analyze variations in the levels of this cytokine in this study (Table 2).

## Discussion

The role of cytokines in the progression of diabetic retinopathy and the formation and maintenance of diabetic macular edema has long been studied.

Molecules such as interleukins, TNF, VEGF, and b-FGF are important components of the pathophysiology of diabetic retinopathy and have been extensively studied to determine their functions in this disease.

In this study, we performed an unprecedented analysis of variations in the levels of these cytokines during diabetic macular edema treatment in patients with and without peripheral retinal ischemia using the antiangiogenic drug ranibizumab.

The purpose of selecting patients with or without peripheral retinal ischemia was to investigate how hypoxia influences the expression of these cytokines and interferes with the outcomes of treatment for diabetic macular edema as well as to analyze the effects on the activity of an anti-VEGF drug.

Knowing the importance of these cytokines, we chose to study their concentrations during treatment for diabetic macular edema with a drug that stabilizes vascular permeability and inhibits one of the studied cytokines.

According to previous studies, the intraocular levels of TNF and IL-1 are elevated under conditions of edema because they can induce an increase in vascular permeability through breakdown of the inner blood–retina barrier^17,19,20^.

In this study, the levels of IL-1 and TNF in Groups 1 and 2 before treatment were slightly higher than those in the control group. In the longitudinal analysis, we observed that the levels of IL-1 and TNF varied little in both groups during the study.

Interleukin 6 is considered a potent proinflammatory cytokine that plays important roles in the mechanisms underlying the chronification of inflammatory processes^23^.

Chernykh and collaborators showed that patients with proliferative diabetic retinopathy had significantly high levels of IL-6 in the vitreous than patients without diabetes^21^. An important aspect of the research conducted by the Chernykh group was the selection of patients with RDP, that is, patients visually diagnosed with retinal ischemia.

In this study, the median values of IL-6 before treatment were approximately 2.5 times higher in ischemic patients than in nonischemic patients and 3.4 times higher in the treatment groups than in the control group at the beginning of treatment. Thus, we hypothesize that this cytokine has a strong relationship with retinal hypoxia.

VEGF is considered one of the main agents in the progression of retinal vascular diseases. VEGF stimulates inflammatory processes and increases vascular permeability and new vessel formation^24–27^.

Osamu and collaborators studied the variation in VEGF concentration in aqueous humor before and after the intravitreal injection of bevacizumab in patients with proliferative diabetic retinopathy^28^. The treatment was administered one week before the patients underwent *pars plana* vitrectomy surgery. The samples were collected before the injection and during surgery. These researchers reported a significant decrease in the concentration of vitreous VEGF one week after the administration of bevacizumab^28^.

Another study also reported data on VEGF concentrations in patients with DR. The results suggested a significant relationship between VEGF levels and disease progression, showing the importance of this signaling protein in the pathogenesis of diabetic retinopathy^29^.

Of all the molecules studied, VEGF showed the greatest variation in concentration, with a decrease of approximately fivefold in both groups (Fig. 8).

Although we observed higher VEGF levels in patients with peripheral ischemia, these values were not significant different from those in patients without peripheral retinal ischemia (Table 1).

The expression of b-FGF is related to the duration of tissue exposure to ischemia. Normally, b-FGF production is increased when there is a prolonged period of ischemia. One of the functions of b-FGF is to stimulate cell proliferation and angiogenesis^30^.

A study published in 2015 analyzed the levels of VEGF and b-FGF in patients with proliferative diabetic retinopathy who were treated with bevacizumab. This previous study included 68 eyes divided into 2 groups: Group 1 patients received bevacizumab treatment 5 or 14 days before vitrectomy; and Group 2 patients had a macular hole without retinopathy or previous treatment with bevacizumab. These researchers concluded that patients with proliferative diabetic retinopathy had higher concentrations of VEGF than those without this disease. Analysis of the concentration of b-FGF revealed that those with a longer interlude from treatment to surgery had higher levels of b-FGF than those in the other groups^31^.

In our study, b-FGF was detected in only 3 patients in Group 1 and none in Group 2. We did not find any justification for the detection of this molecule in only Group 1. In Group 2, the duration of ischemia or the presented neovascular condition may not have been sufficient to induce the production of this cytokine (Table 1).

## Conclusions

1. Patients with diabetic macular edema without peripheral retinal ischemia detected by wide-angle fluorescein angiography showed a significant increase in the median level of interleukin 6 and a significant decrease in VEGF level after treatment with ranibizumab. No significant variations were detected in IL-1, IL- 8, IL-10, IL-12, b-FGF, or TNF.
2. Patients with diabetic macular edema with peripheral retinal ischemia detected by wide-angle fluorescein angiography presented a significant variation in only VFGF levels after treatment with ranibizumab. IL-1, IL-6, IL-8, IL-10, IL-12, b-FGF, and TNF did not show statistically significant variations.

## Data Availability

April 2018 to December 2018.
